# Data on regulation of the gene for the adipocyte-enriched micropeptide Adig/Smaf1 by qPCR analysis and luciferase reporter assay

**DOI:** 10.1016/j.dib.2016.09.034

**Published:** 2016-09-24

**Authors:** Gang Ren, Nicholas Cairl, Ji Young Kim, Cynthia M. Smas

**Affiliations:** University of Toledo College of Medicine, Department of Biochemistry and Cancer Biology and the Center for Diabetes and Endocrine Research, Toledo, OH, USA

**Keywords:** Adipocyte, Adipose tissue, PPARgamma, Nutritional regulation, Obesity, Adipogenesis

## Abstract

This article describes qPCR analysis for the Adig/Smaf1 gene in multiple *in vitro* adipocyte differentiation models including white and brown adipogenesis, cell lines and primary cultures. The article also contains qPCR data for transcript levels of Adig/Smaf1 in a wide panel of murine tissues. Expression of Adig/Smaf1 transcript in white and brown adipose tissue in fasted and refed mice is reported and also data for Adig/Smaf1 transcript expression in genetically obese *ob/ob* mice. Data on the effects of siRNA-mediated knockdown of Srebp1c on Adig/Smaf1 transcript levels in 3T3-L1 adipocytes are shown. Luciferase reporter assays provide data for regulation of an ~ 2 kb fragment of the 5′ flanking region of Adig/Smaf1 gene by PPARγ/RXRα. This data is related to a research article describing Adig/Smaf1 protein expression, “Expression, regulation and functional assessment of the 80 amino acid Small Adipocyte Factor 1 (Smaf1) protein in adipocytes” (G. Ren, P. Eskandari, S. Wang, C.M. Smas, 2016) [1].

**Specifications Table**

**Table:** 

Subject area	*Biology*
More specific subject area	*Metabolism,Gene Expression, Adipose Tissue, Cell Differentiation*
Type of data	*Graphs, Figures*
How data was acquired	*qPCR studies and luciferase assay*
Data format	*Analyzed*
Experimental factors	*Adipogenesis, isolation of adipose tissues and liver for qPCR analysis.*
	*Transfection of siRNA and expression vectors in cultured cells for qPCR analysis or luciferase assay.*
Experimental features	*Adipogenesis of 3T3-L1 preadipocytes and of other cell culture models of in vitro adipocyte differentiation was induced using standard methods. Tissues were harvested from C57BL/6 mice and from genetically obese ob/ob mice. RNA was extracted from cells and tissues by Trizol method, and cDNA was synthesized and utilized for qPCR studies. Hela cells were transfected with an Adig/Smaf1 luciferase reporter construct in the absence or presence of co-transfection of PPARγ and RXRα expression constructs to determine transactivation of the Adig/Smaf1 promoter region using luciferase reporter assay.*
Data source location	*Toledo, OH*
Data accessibility	*All data is available within the article.*

**Value of the data**

• The Adig/Smaf1 gene encodes an adipose-enriched micropeptide of 80 amino acids [1] and there is a growing interest in the functions of proteins encoded by small open reading frames (sORFs) [2].
• The Adig/Smaf1 gene was recently implicated in the regulation of human leptin levels based on GWAS data [3].
• The function of Adig/Smaf1 remains unknown, therefore quantitative data on regulation of the Adig/Smaf1 gene may provide insight to its metabolic or other roles.
• The data can be compared and contrasted with data for other adipocyte genes or genes induced in fatty liver of obese mice.
• The data provides a foundation for additional studies of how PPARγ regulates the Adig/Smaf1 gene.

## 1 Data

Here we significantly extend the limited information available to date for transcript expression for Adig/Smaf1 in adipose cells and tissues, previously determined by this laboratory [4] and by others [5]. This includes graphical qPCR data for expression of this gene during preadipocyte to adipocyte conversion using multiple *in vitro* adipogenesis models and with obesity and nutritional status in murine tissues. We also report data on regulation of Adig/Smaf1 gene expression by transcription factors PPARγ and Srebp1c using siRNA and/or luciferase reporter assay.

## 2 Experimental design, materials and methods

### 2.1 Cell culture, *in vitro* adipocyte differentiation and treatments

Methods for culturing and differentiating the various cell lines utilized in this study have been previously described [1], [6], [7].

### 2.2 Animal studies

**Fig. 1:**
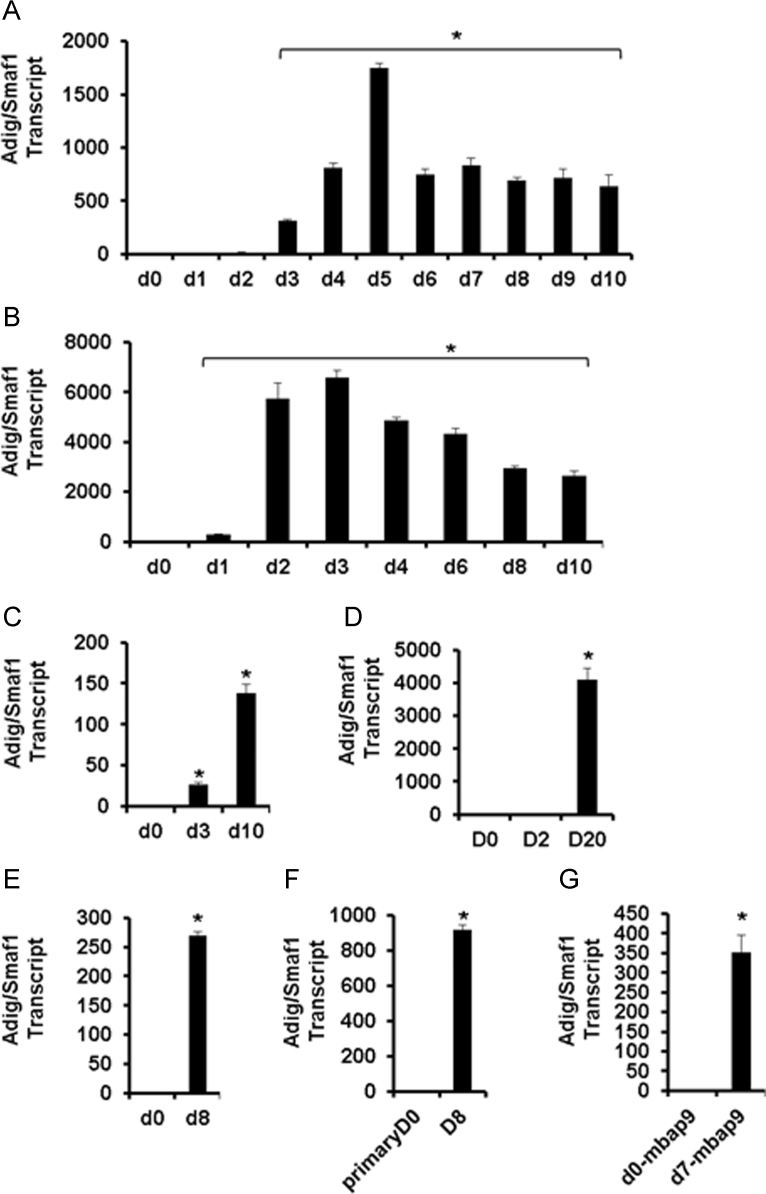
Data on qPCR for Adig/Smaf1 transcript level in multiple *in vitro* adipogenesis models. A. 3T3-L1; B. WT-BAT; C. ScAP23; D. C3H10T1/2; E. Ad3.7. F. Primary murine preadipocytes before (D0) and after (D8) differentiation. G. mBAP-9 cells. D indicates days post induction of differentiation and the D0 value was set to 1. For *A* and *B*, * indicates *p*<0.05 for each of the individual bracketed columns *vs.* d0. For *C–G*, * indicates *p*<0.05 for designated column compared to D0, and in the case of D, compared to D0 or D2.

**Fig. 2:**
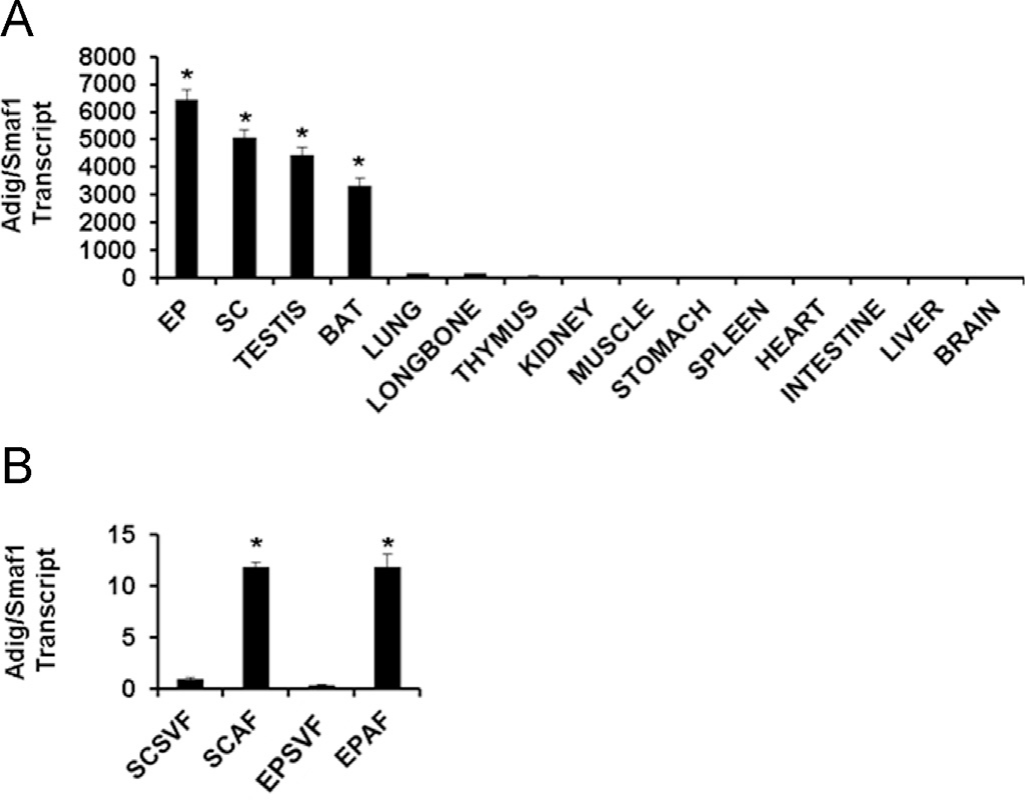
Data on qPCR analysis for expression of Adig/Smaf1 transcript in murine tissues and primary cells. A. In a panel of murine tissues. EP, epididymal WAT; SC, subcutaneous WAT; BAT brown adipose tissue. *p*<0.05 for each of the columns with an asterisk, compared to each column not designated with an asterisk. The value in brain was set to 1. B. Expression of Adig/Smaf1 transcript in fractionated whole EP or SC WAT. SVF, stromal vascular fraction; AF, adipose fraction. An * indicates *p*<0.05 compared to respective SVF fraction column; the value in the SCSVF column was set to 1.

Studies were in strict accord with the guidelines in the Guide for the Care and Use of Laboratory Animals of the NIH. For Figs. 1 and 2 data of Adig/Smaf1 transcript expression in wild type C57BL/6 and *ob/ob* mice tissues, and for fasting and refeeding, are as described [7], [8].

### 2.3 RNA preparation and qPCR

**Fig. 3:**
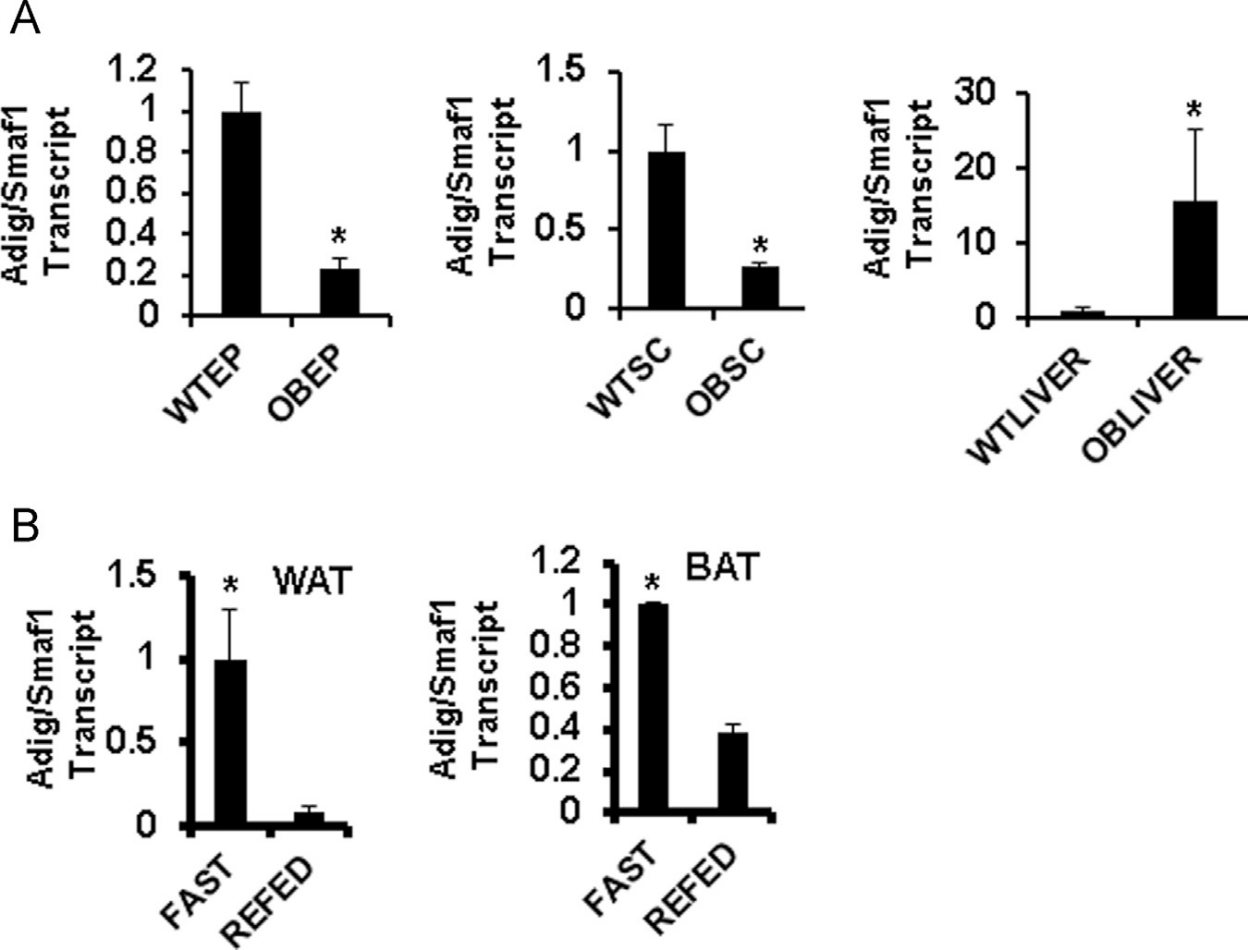
Data on qPCR analysis for expression of Adig/ Smaf1 transcript in murine obesity and under nutritional manipulation. A. In wild type (WT) or *ob/ob* (OB) mouse tissues for epididymal (EP) and subcutaneous (SC) adipose tissue and in liver. B. Regulation of Adig/Smaf1 expression in white adipose tissue (WAT) and brown adipose tissue (BAT) with 18 h fasting (fast) or fasting followed by 8 h of refeeding (refed). An * indicates *p*<0.05 compared to WT (for panel A) or to refed (for panel B).

**Fig. 4:**
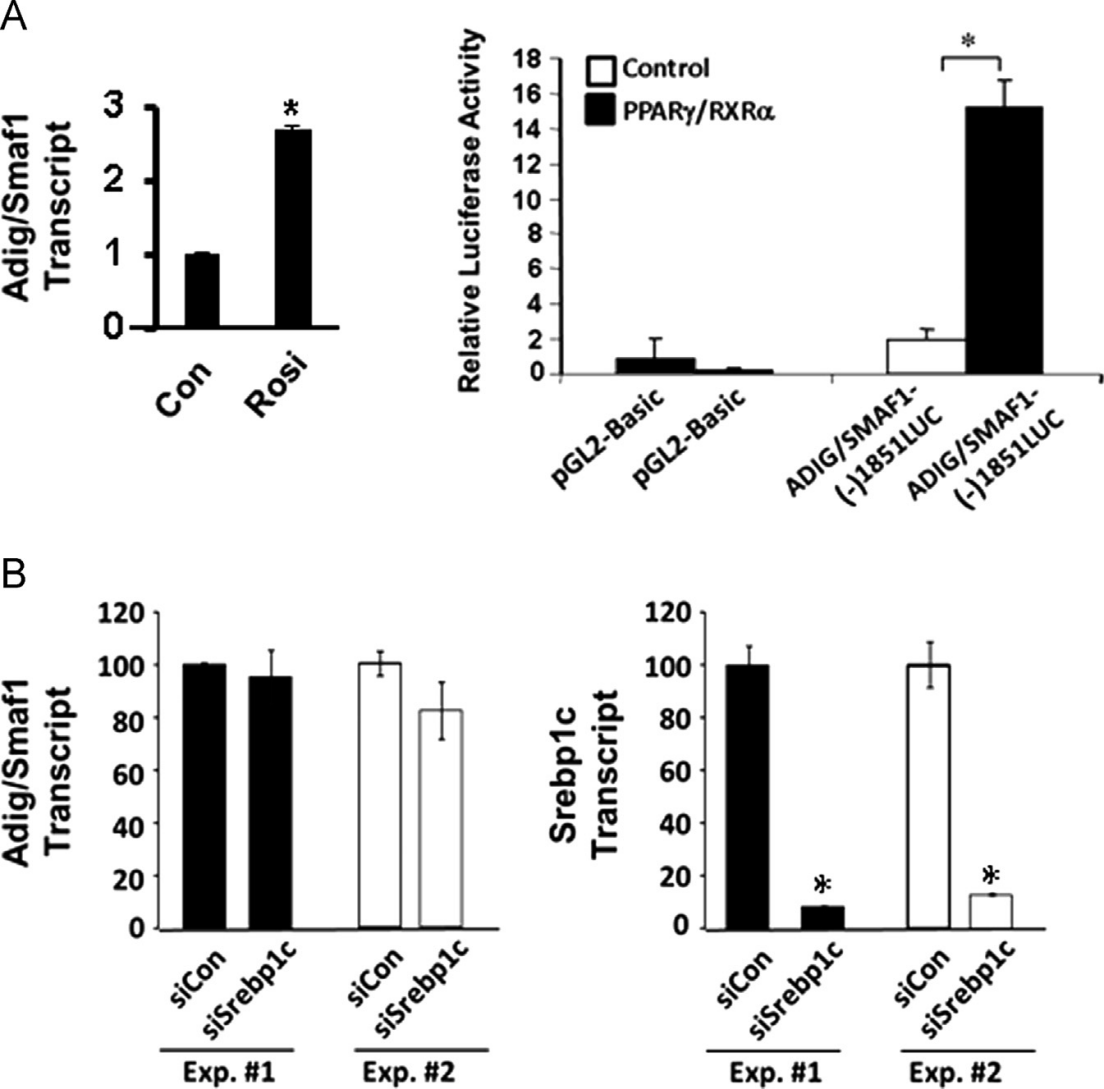
Data on expression of Adig/Smaf1 by PPARγ and Srebp1c. A. Left Panel: qPCR for 3T3-L1 adipocytes treated with vehicle (Con) or with the PPARγ ligand rosiglitazone (Rosi) for 24 h. Right Panel: Luciferase reporter assays for regulation of Adig/Smaf1 promoter region. Hela cells were transfected with the (−)1851 Adig/Smaf1 luciferase reporter construct in the presence or absence of co-transfection with PPARγ and RXRα plus PPARγ ligand. B. qPCR analysis of Adig/Smaf1 and Srebp1c transcript levels in 3T3-L1 adipocytes transfected with a control siRNA (siCon) or with siRNA for Srebp1c. An * indicates *p*<0.05.

For Fig. 1, Fig. 2, Fig. 3, Fig. 4, RNA was extracted from cultured cells and mouse tissues, further processed for qPCR, and qPCR carried out and analyzed as previously described [4], [7].

### 2.4 siRNA studies

siGENOME siRNA was from Dharmacon and used at 25 nM. Control was non-targeting siRNA catalog #D-001210-01-05 (siCon). siRNA was electroporated into 3T3-L1 adipocytes as described [7] and samples harvested for qPCR two days, Fig. 4.

### 2.5 Luciferase reporter assay

To prepare luciferase reporter constructs containing the Adig/Smaf1 5′ flanking region, phage DNA for murine Adig/Smaf1 genomic clones was amplified by PCR. Primers were designed from murine Adig/Smaf1 genomic flanking sequence at the Ensembl database (www.ensembl.org) and using sequence data we attained by 5′ RACE analysis for the murine Adig/Smaf1 gene. PCR product containing up to position (−)1851 of the murine Adig/Smaf1 gene was cloned into pGL2-Basic vector (Promega, Madison, WI). Transient transfection of HeLa cells was with Lipofectamine 2000. As indicated in Fig. 4, transfections utilized indicated combinations of PPARγ/RXRα, pGL2-Basic, and Adig/Smaf1(-)1851LUC DNA constructs. All samples were also co-transfected with pRL-null vector, expressing *Renilla* luciferase, as an internal control. At 48 h posttransfection, cultures were treated with 10 μM of the PPARγ ligand 15-deoxy-Δ^12,14^-prostaglandin J_2_ (Caymen Chemical, Ann Arbor, MI). Cell lysates were harvested 24 h later and further analyzed as described [8]. Statistical analyses were conducted using single factor ANOVA.

## Funding source

Funded in part by NIH, United States grant 5R21DK66055 to C.M. Smas and by a grant from University of Toledo Center for Diabetes and Endocrine Research.
